# The Role of Thalamic Population Synchrony in the Emergence of Cortical Feature Selectivity

**DOI:** 10.1371/journal.pcbi.1003418

**Published:** 2014-01-09

**Authors:** Sean T. Kelly, Jens Kremkow, Jianzhong Jin, Yushi Wang, Qi Wang, Jose-Manuel Alonso, Garrett B. Stanley

**Affiliations:** 1 Coulter Dept. of Biomedical Engineering, Georgia Institute of Technology, Emory University, Atlanta, Georgia, United States of America; 2 Department of Biological Sciences, State University of New York, College of Optometry, New York, New York, United States of America; 3 Department of Biomedical Engineering, Columbia University, New York, New York, United States of America; Université Paris Descartes, Centre National de la Recherche Scientifique, France

## Abstract

In a wide range of studies, the emergence of orientation selectivity in primary visual cortex has been attributed to a complex interaction between feed-forward thalamic input and inhibitory mechanisms at the level of cortex. Although it is well known that layer 4 cortical neurons are highly sensitive to the timing of thalamic inputs, the role of the stimulus-driven timing of thalamic inputs in cortical orientation selectivity is not well understood. Here we show that the synchronization of thalamic firing contributes directly to the orientation tuned responses of primary visual cortex in a way that optimizes the stimulus information per cortical spike. From the recorded responses of geniculate X-cells in the anesthetized cat, we synthesized thalamic sub-populations that would likely serve as the synaptic input to a common layer 4 cortical neuron based on anatomical constraints. We used this synchronized input as the driving input to an integrate-and-fire model of cortical responses and demonstrated that the tuning properties match closely to those measured in primary visual cortex. By modulating the overall level of synchronization at the preferred orientation, we show that efficiency of information transmission in the cortex is maximized for levels of synchronization which match those reported in thalamic recordings in response to naturalistic stimuli, a property which is relatively invariant to the orientation tuning width. These findings indicate evidence for a more prominent role of the feed-forward thalamic input in cortical feature selectivity based on thalamic synchronization.

## Author Summary

While the visual system is selective for a wide range of different inputs, orientation selectivity has been considered the preeminent property of the mammalian visual cortex. Existing models of this selectivity rely on varying relative importance of feedforward thalamic input and intracortical influence. Recently, we have shown that pairwise timing relationships between single thalamic neurons can be predictive of a high degree of orientation selectivity. Here we have constructed a computational model that predicts cortical orientation tuning from thalamic populations. We show that this arrangement, relying on precise timing differences between thalamic responses, accurately predicts tuning properties as well as demonstrates that certain timing relationships are optimal for transmitting information about the stimulus to cortex.

## Introduction

Sensory systems serve the purpose of allowing us to extract perceptually relevant features from the environment. Although there are certainly examples of sensory features whose coding originates in the sensory periphery (e.g. auditory frequency, visual color, etc.), the more intriguing and less well understood phenomena involve the emergence of feature selectivity in more central brain structures that do not just inherit the selectivity from the periphery. Perhaps the most well studied of these phenomena is that of orientation selectivity in primary visual cortex (V1), where many if not most neurons in the mammalian primary visual cortex exhibit differential firing activity for visual stimuli at different orientations, despite the fact that the neurons projecting from the lateral geniculate nucleus (LGN) serving as input to V1 exhibit little to no orientation preference on their own [1] (see [2] for a review). This implies that the thalamocortical link is a transformative location for representation of stimuli as collections of particular features rather than samples (i.e. it does far more than simply relay luminance values to the cortex). This transformation can serve as a general model for how sensory systems convey increasing feature selectivity as the information moves to higher-order brain areas. How do these convergent thalamic structures drive cortical feature selectivity, and in what way do populations drive this selectivity?

The mechanistic origin of orientation tuning in V1 has been vigorously explored in the literature [1]–[5]. In their seminal work, Hubel and Wiesel outlined a conceptual model that involved the projection of LGN neurons along a particular axis of orientation to a common cortical target [1], the core connectivity of which was subsequently confirmed in recordings from connected pairs of neurons in LGN and V1 [6]–[8]. Although the relative roles of this feedforward architecture versus cortico-cortico connectivity in sharpening and refining orientation selectivity in such phenomena as contrast-invariance and cross-orientation suppression has been intensely debated [2], [9], the thalamic basis for the origin of the basic selectivity is not in dispute, and by its nature implies a role for the timing of thalamic inputs to the cortical target. That is, the several decade old proposal by Hubel and Wiesel conceptually suggests that an edge activating the subset of thalamic neurons projecting to a common cortical target at the same time would naturally drive the cortical neuron more so than when the thalamic inputs are activated at different times, establishing the orientation tuning for the cortical neuron. However, the precise role of timing of thalamic inputs in the downstream cortical orientation selectivity is not known. In the context of the natural visual environment, it has been shown that LGN neurons (individually and across pairs) are temporally precise to a time scale of 10–20 ms, a level that is matched to what is necessary to capture the timescale of changes exhibited in natural scenes [10]–[12]. Further it has been demonstrated that neurons in the primary visual cortex are extremely sensitive to short intervals between incoming thalamic spikes also on the time scale of approximately 10 ms [13]–[22] and that common cortical convergence is most probable when receptive fields overlap [7], [13]. All of these findings collectively suggest that feature selectivity is likely to arise from the modulation of precise timing among overlapping populations of neurons in LGN and that this modulation drives the coactivation of neurons within the populations. Finally, we have recently shown that considering just the coactivation between pairs of electrophysiologically recorded thalamic neurons reveals in many cases extremely sharp orientation tuning even when the receptive fields are highly overlapped [23].

Here, to explore the role of the precise timing of thalamic spiking in the orientation tuning of the downstream cortical neurons to which the thalamus projects, we utilized experimental population recordings of single units from the LGN region of the visual thalamus in concert with a large-scale thalamocortical model. Specifically, based on anatomical and physiological evidence concerning the convergence of thalamic input to cortical layer 4, we constructed thalamic sub-populations from experimentally recorded thalamic spiking in response to oriented visual stimuli, and systematically controlled the precise timing across the sub-population and its direct impact on the downstream orientation tuning. We found that the conventionally measured tuning sharpness was remarkably invariant over a wide range of peak LGN timing precisions, but the trial-to-trial variability in cortical response was strongly influenced by the timing precision of the LGN inputs. From a decoding perspective of an ideal observer of the cortical response, this complex relationship led to a decreasing error in estimation of orientation with increasing thalamic precision, and a corresponding increase in the information rate, both saturating for peak thalamic precisions of 10–20 ms, a finding which was invariant to the overall width of cortical orientation tuning. Taken together, the results here provide a compelling picture for the role of stimulus-driven thalamic synchrony in the emergence of cortical feature selectivity.

## Results

### Spatial Distribution of LGN Populations

Neurons in layer 4 of primary visual cortex are driven by sub-populations of projecting LGN neurons with receptive fields that are highly overlapped, thus representing a relatively limited area of visual space [24]. Although individual LGN neurons are relatively insensitive to the orientation of drifting sinusoidal gratings, the synchrony across neuron sub-populations is often highly sensitive to the orientation, a product of the relative spatial geometry of the receptive fields and the underlying temporal dynamics of component neurons [23]. LGN populations which share a convergent cortical neuron are both large (approximately 30 neurons [8]) and highly overlapped. Since it is not currently possible to record from such dense and numerous clusters in the LGN, we implemented a population-filling method to quantify the synchronization properties of the sub-population. Specifically, in the population-filling method we utilized simultaneous recordings of spiking activity of small sub-populations of LGN neurons whose receptive fields span a small area of visual space (see Methods).

**Figure 1:**
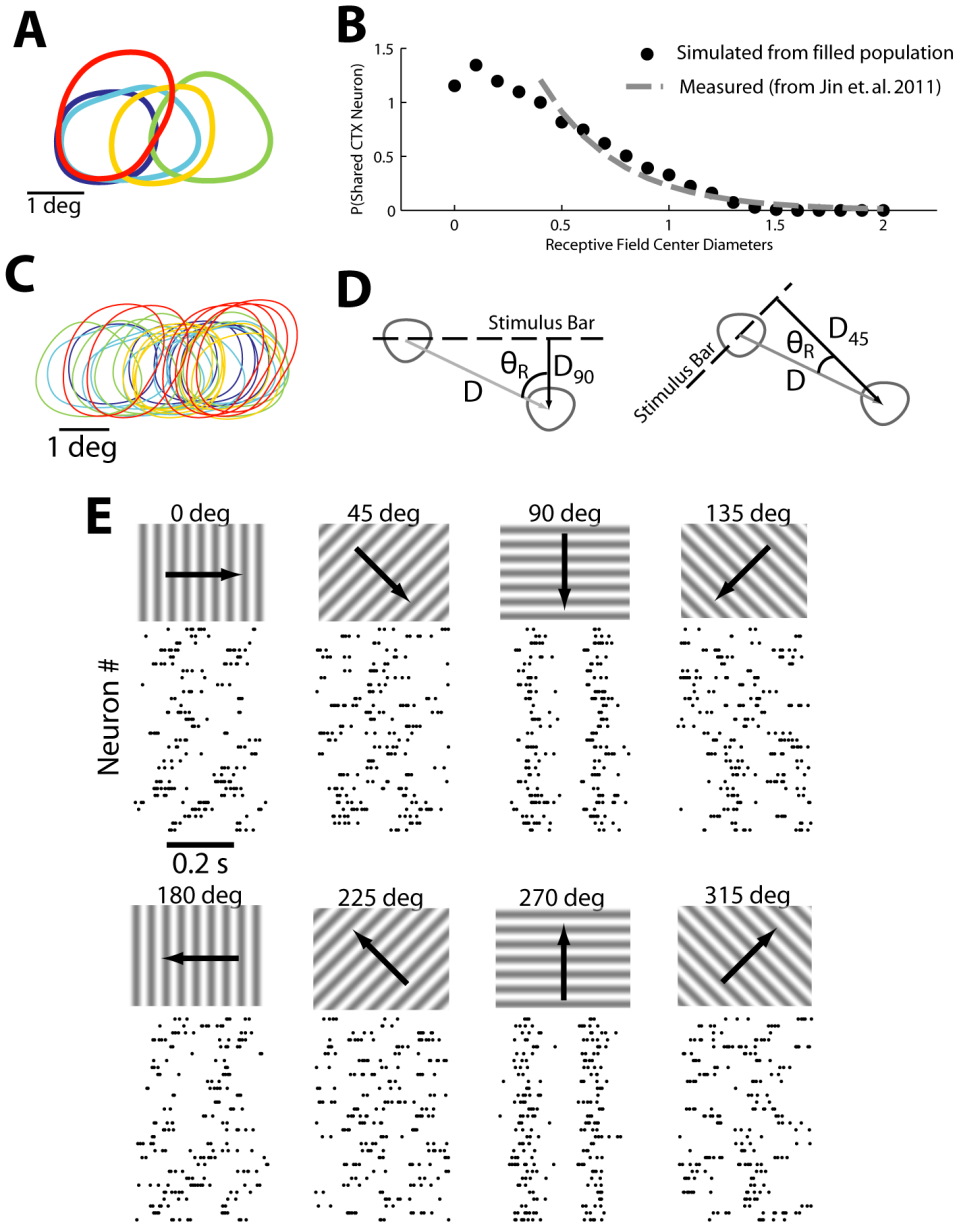
Filling in population from recorded neuron receptive fields. **A**. The original simultaneously recorded receptive fields of 5 neurons. **B,C**. The original receptive fields were duplicated and randomly shifted so that the resulting population (**C**) matched the previously measured distribution of RFCD values (**B**) Solid circles indicate RFCD measures from the population in **C**, while the dashed line indicates the expected distribution (see Methods). **D**. The spatial shift in each receptive field describes a particular distance perpendicular to the stimulus orientation that each receptive field shifts; using the spatial and temporal frequencies of the stimulus this can be translated into a timing shift. **E**. Once spike times are appropriately shifted for each neuron in the population, rastergrams reveal spiking alignment only for 90 and 270 degree stimulus orientations.

Single unit activity was collected in response to spatiotemporal white noise, and receptive fields (RFs) were mapped using standard spike-triggered averaging (see Methods). The RFs of a pool of simultaneously recorded LGN neurons are shown in Figure 1A, where the RF for each neuron is represented as the 20% contour. Note that in this recording, 5 neurons were recorded simultaneously, where each of these neurons is represented as a different color in the figure. We have previously provided experimental measures of the distribution of receptive field spacing of pairs of LGN neurons monosynaptically connected to a single cortical cell [8] and populations of LGN neurons to a single cortical orientation column [24], as shown with the dashed gray curve in Figure 1B. Specifically, this measure provides a probability distribution of the distances between receptive fields, as measured by the distance between the RF centers normalized by the diameter of the larger of the two RFs, referred to here in units of receptive field center diameter (RFCD) - see [24].

From experimental data in [24], the distribution of separations was modeled as 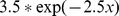, where x is the separation in units of RFCD, which is described only for the range of 0.4 to 2.0. Using the neurons in Figure 1A as templates and the relationship in Figure 1B (dashed line) as a rule, we filled out the assumed remainder of the population by translating the receptive fields in visual space, creating a dense and accurate convergent LGN population, as shown in Figure 1C. The receptive field centers were randomly shifted such that the amount of visual space covered did not change relative to the visual space covered by the original simultaneously recorded population. This method resulted in a distribution of RF separations consistent with previous experimental findings (simulated distribution shown with solid black circles, Figure 1B). Note that because the original population was itself elongated in the horizontal axis, the resultant shifts for this population were also mostly horizontal although some receptive field locations also moved vertically. The resultant cluster of receptive fields would be typical for a population that has a major and minor axis as opposed to being more circularly arranged. The resulting aspect ratio of the cluster of RFs in Figure 1A is approximately 2.4∶1, when measured as the ratio of the longer dimension to the shorter dimension of the area covered by the RF contours. It is important to note that this aspect ratio is lower than the majority of existing models [1], [3]–[5], where aspect ratios range from 3 to 4 (but see [5] for a much smaller aspect ratio).

Spiking activity was also collected in response to drifting sinusoidal gratings (0.5 cycles/degree, 5 Hz, 100% contrast - see Methods). The individual LGN neurons had mean firing rates that ranged from 16 to 28 Hz which were relatively insensitive to the stimulus orientation. To generate the population activity in response to the drifting gratings, we utilized the spatially translated RFs as described above, and imposed temporal shifts in the spiking activity based solely on the geometry related to the RF centers, as illustrated in Figure 1D. Specifically, a spatial translation of the RF by x degrees horizontally and y degrees vertically imposes a latency shift of the neural response by an amount proportional to the component of the vector connecting the centers of the two RFs orthogonal to the edge of the drifting grating, scaled by the speed of the drift (see Methods). For the collected datasets, spiking activity was collected at each of eight drifting directions with sinusoidal gratings. For each stimulus condition, each randomly placed neuron was assigned a random trial from the original neuron from which it was derived and the shift latency value was added to all spike times in the chosen trial. In this spirit, we view the trial to trial variability in spiking activity for a single neuron as representative of the across neuron variability on a single trial. The resulting population response at each orientation is shown in Figure 1E. For most orientations, spike times within the population uniformly distributed across the entire trial timespan. However, at 90 and 270 degrees, the spike times line up rather precisely between all neurons in the population, reflecting a high degree of synchrony at these orientations.

### Physiological Timing Jitter

**Figure 2:**
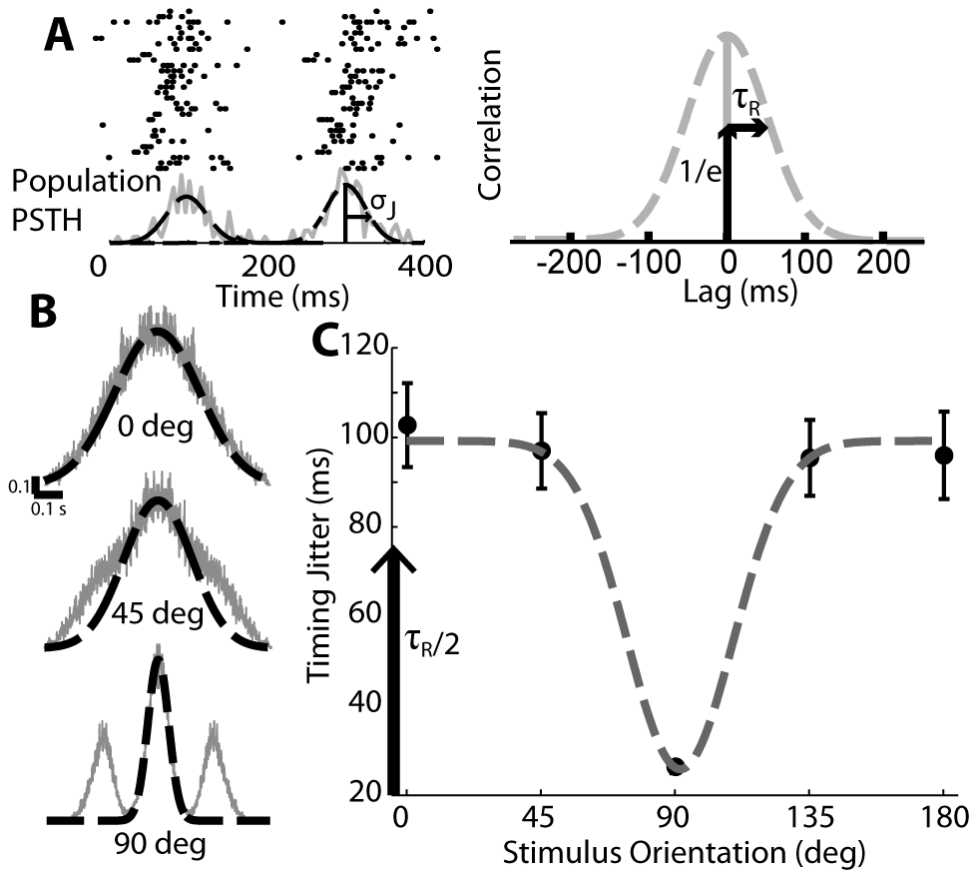
Timing jitter is defined by the spike-time auto-correlation width. **A**. Spike timing auto-correlations come from the spike times across the entire population, collapsed to a single spike train. This can be represented by a PSTH with a particular defined width 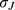 which represents timing jitter in ms. The resultant auto-correlation also has a defined width 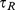 and this value is the lag at which the auto-correlation is equal to 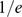, assuming the auto-correlation is appropriately normalized. By construction, 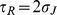 (see Methods). **B**. Example spike time auto-correlation widths (fit to −0.1 s to 0.1 s with a Gaussian) at non-preferred, moderately preferred, and highly preferred orientations (top to bottom). **C**. The timing jitter is defined through the width of these Gaussian fits and decreases as the stimulus orientation nears the preferred orientation. Black circles indicate measurements taken from recorded data and arranged as in Figure 1, and the dashed gray line indicates a Gaussian fit. Error bars are standard deviation over multiple simulations of the population.

The degree of synchrony across this population of neurons is a function of the orientation of the drifting gratings, as well as the variability in spiking timing across neurons within the population. To quantify the synchrony, we used a timing jitter metric, which utilizes the width of the spike-time auto-correlation computed from all spikes in the population (roughly equivalent to the PSTH width). A brief overview of how the auto-correlation is calculated is demonstrated in 2A. The collection of spike times across the input population is collapsed into a single spike train, which represents all the projecting thalamic input on the cortical target neuron. This spike train is then used to calculate all of the pair-wise timing differences between every input spike in the population, the histogram of which forms the auto-correlation estimate. There are two values of interest: the population PSTH (with a width of 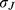) and the “response timescale” of the auto-correlation function (given by 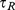). These related values provide us with an approximation for the synchronization within the neural population. When synchrony is high, the spike time auto-correlation has a narrow width and thus there is little jitter. Alternatively, when synchrony is low, the auto-correlation has an increased width and jitter is very high, a property that is demonstrated in Figure 2B. From top to bottom in the figure, the level of synchrony in the population increases, spike times become more clustered, and the auto-correlation has a correspondingly decreasing width. Note that each auto-correlation covers the lag range from −400 ms to +400 ms. Each auto-correlation function was fit with a Gaussian between −100 and +100 ms to eliminate any effects of periodicity in response to the drifting sinusoidal grating. The corresponding width of this Gaussian fit was then utilized as the measure of timing jitter. As in [10], the timing jitter was defined as the half the latency at which the Gaussian fit is equal to 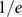 (see Methods and Figure 2A). The timing jitter of the population is shown as a function of the stimulus orientation in Figure 2C, where the random sampling of single trials of the template neuron was repeated 50 times. At the most asynchronous stimulus orientations (in this case perpendicular to the elongated axis of the RFs of the population), the timing jitter was approximately 100 ms. At the preferred orientations, when synchrony was maximized, the timing jitter was approximately 24 ms. The timing jitter as a function of stimulus orientation was fit with a Gaussian function (gray dashed line in Figure 2C) and exhibited a characteristic tuning width of approximately 31 degrees (standard deviation), a finding which was consistent for two of the three animals. In the third animal there was an insufficient number of strongly-driven neurons with identical polarities (ON- versus OFF-center) to allow for a reasonable reconstruction of a population with more than 2 or 3 neurons. With so few neurons, the population displayed more and more properties of the response of a single neuron as opposed to a rough average of multiple neurons and the overall orientation tuning decreased as the population approached the orientation-agnostic response properties of a single input neuron. To determine the generality of our findings here, we utilized other metrics from previously published studies, with a focus on the reliability method used in [25] which is easily adaptable to population data. We found that qualitatively the results were similar to our own findings; just as jitter decreases in our sample population at 90 and 270 degrees (Figure 2C) the reliability across all the neurons in the population is significantly higher at 90 and 270 degrees. We thus expect that the synchronization observed across all neurons in the population is not affected by the metric chosen to measure it.

By construction, the degree of synchrony across the population of neurons in Figure 1D is a function of the orientation of the drifting gratings and across neuron variability in spiking, independent from geometry. The across neuron variability in timing thus set the lower bound of timing jitter in Figure 2C. To more fully explore the role of synchrony in shaping the feature selectivity in the downstream cortical response, we effectively replaced the across-neuron variability in spike timing with variability under our control. Specifically, we utilized a single trial spike-train for a template neuron and introduced the latency associated with the translation of the receptive field as in Figure 1D, but subsequently added variability to each spike time in the form of a Gaussian random variable with zero mean and variance 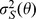. So long as the population firing rate reaches a particular minimum mean level it does not matter which template neuron is chosen to provide the spike train; we found that nearly all neurons from all three animals provided consistent simulations of cortical activity. Using a single trial has the effect of removing the effects of variable spike count across trials for a particular neuron in addition to providing the exact control over the timing jitter. Of key importance is the value 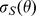, which is the stimulus-dependent component of timing jitter (see Methods for expanded description). This value is related to but not equal to the timing value measured from the full populations; 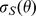 represents the underlying stimulus-based modulations to synchrony that give rise to the full timing jitter relationship shown in Figure 2C. This timing variability quantity 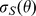 was parameterized as a Gaussian function of 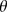 and was manually tuned to reproduce the population timing variability curve in Figure 2C. From here on out, when we refer to “minimum timing jitter” we are referring to the minimum value of 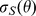 that occurs at the preferred orientation.

### Cortical Orientation Tuning

**Figure 3:**
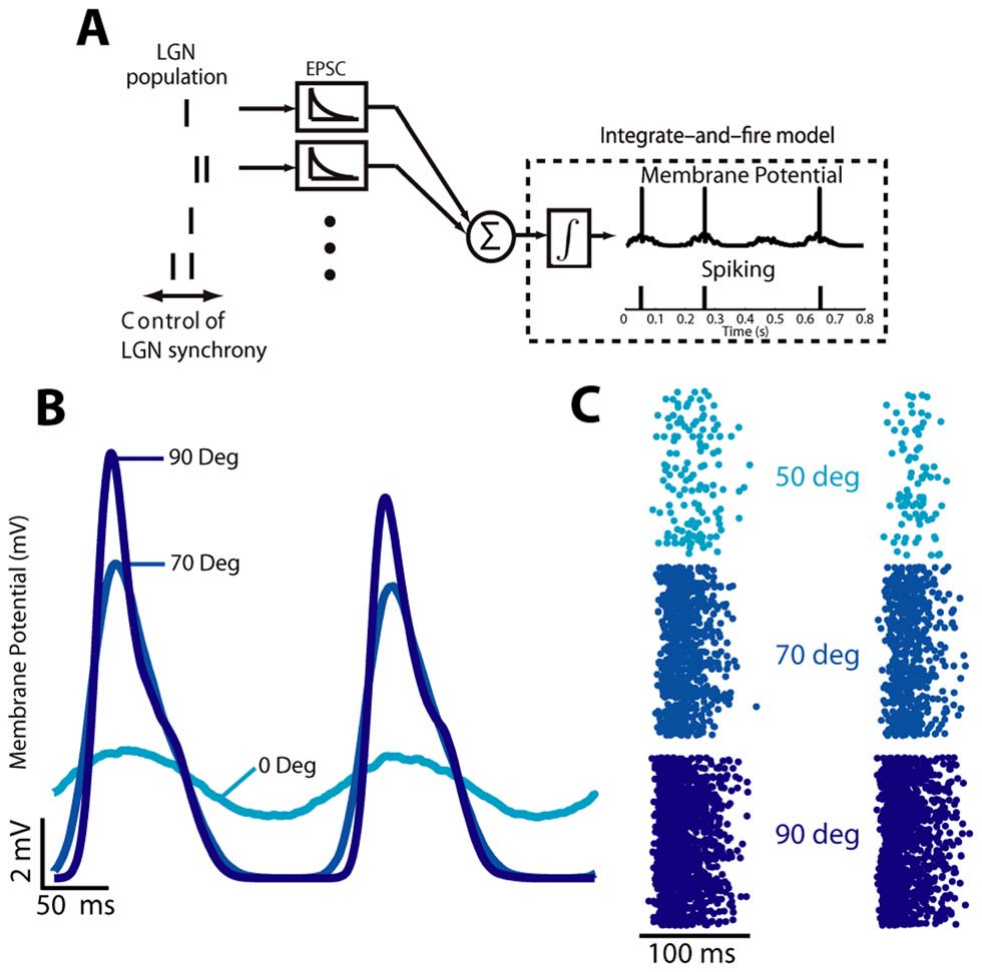
Model and simulated output characteristics. **A**. The model imposes simple control over input spike synchrony and uses a leaky integrate-and-fire construction to determine membrane potential and output spike times. **B**. The simulated cortical membrane potential has an amplitude that is strongly affected by the stimulus orientation, but also a mean value that changes with orientation due to reset characteristics. **C**. Orientations which are closer to the preferred orientation produce dramatically increased numbers of spikes.

To determine how different levels of input synchrony affect the downstream cortical response and the corresponding feature selectivity, we simulated the cortical layer 4 neuron response to the drifting gratings at different orientations. The previously described populations were used as input to this model, modulating the minimum value of 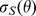 to cover a range of 6 to 40 ms of population timing jitter. To model the cortical neuron, we used a biophysically inspired integrate and fire model — illustrated in 3A — that generates a continuous membrane potential and corresponding firing activity, similar to that in [22] and [23] - see Methods. In brief, the model lumps all input spike times together in a common spike train, laying down a superimposed EPSC for each input spike (all of which thus have equal weighting). This model is represented by the differential equation
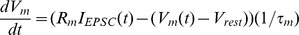
with a fixed parameter set to determine the point by point membrane potential and with a fixed time step of 0.05 ms. Membrane potential traces show a clear stimulus-driven modulation [26]–[28] that increases in amplitude towards the population's preferred orientation when averaged over 1000 trials, as shown in Figure 3B. Single trial responses, with the exception of the nonphysiological mechanics of the hard reset, match typical recordings from cortical neurons using examples from Carandini & Ferster [29] as a primary source for comparison. Further, the tuning properties (firing rate and tuning half-width at half-height) match reported values, as will be shown later. The reset mechanics did not adversely affect the accuracy of the results as the spiking statistics and tuning curves were consistent with experimental observations. Cortical spike counts, as shown in Figure 3C rastergrams, increased dramatically as the stimulus approached the preferred orientation, and the underlying stimulus driven events became very clear. Again, these spike count rastergrams are representative of what would be expected from cortical neurons, although this is easier to see in the cortical tuning curves.

**Figure 4:**
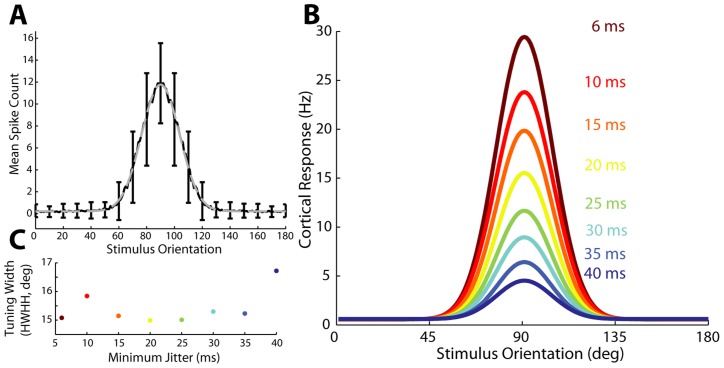
Tuned output of cortical model. **A**. Example tuning curve (black line) at 6 ms of minimum jitter is fit very well by a Gaussian curve (gray dashed line). Standard deviation is illustrated at 10 degree increments, revealing sometimes significant variance in output spike count. In general this reflects the variability of the input spike counts. **B**. The integrate and fire cortical model outputs tuning curves that are well-described by a Gaussian model with an amplitude that decreases with increasing minimum jitter (dark red: 6 ms, dark blue: 40 ms). **C**. The tuning width varies over a small range across the entire range of minimum jitter values simulated.

By construction of the thalamic input, the model generated cortical responses that exhibited orientation selectivity. Although the original experimental data was collected only for 8 grating orientations, the parameterized construction described in Figure 2 allowed simulation at an arbitrarily fine grain (chosen to be at 1 degree increments here). The resulting mean cortical firing rate across all orientations for a minimum jitter of 6 ms is shown in Figure 4A, which is stereotypical of recorded responses of neurons in the primary visual cortex [29], with higher firing rates possible when using different neurons for thalamic spike times. The cortical firing rate as a function of stimulus orientation was fit with a local Gaussian over a 180 degree span, as shown with the dashed curve. The parametric fits for each of a range of minimum jitter cases are shown in Figure 4B. The colors indicate decreasing levels of synchrony with dark red representing high synchrony (6 ms of jitter) and dark blue representing low synchrony (40 ms of jitter). The overall magnitude of the cortical response decreased with increasing amounts of jitter, as reflected in the overall amplitude of the tuning curves. The sharpness of orientation tuning is quantified though the half-width at half-height (HWHH) of the tuning curve [29], [30]. Consistent with reported values for firing rate, the HWHH tuning width for firing rate was approximately 15 to 16 degrees and was relatively insensitive to the LGN input synchrony (Figure 4C) up until 35 ms of input jitter at which point the tuning width increases by approximately 1.5 degrees. These values are on the lower end of expected tuning widths [9], [29], [30]. Carandini & Ferster [29] noted that due to experimental limitations they cannot discriminate half-widths less than 17 degrees, a value that they find for almost all recorded neurons. On the other hand different studies [31], [32] have reported tuning widths with significant numbers of neurons with small (10–15 degree) tuning widths. Note that the primary results of the analysis were relatively invariant to the actual tuning width, as we will demonstrate later.

### Statistics of Orientation Tuning

**Figure 5:**
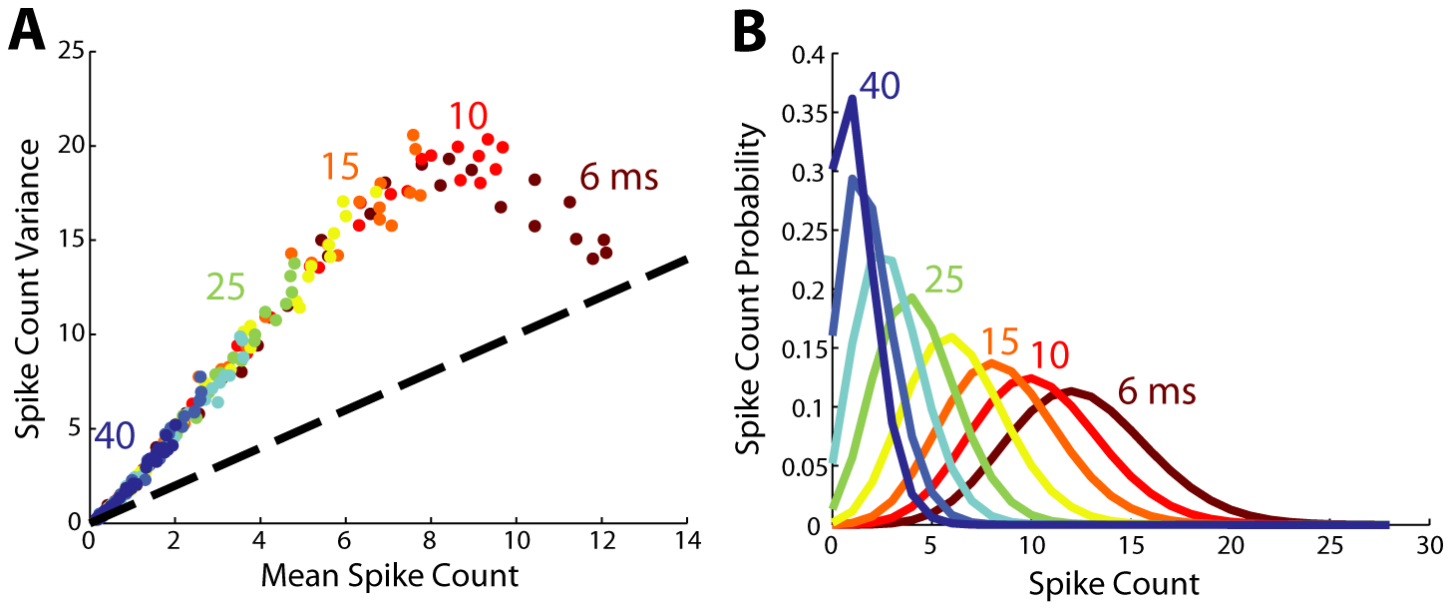
Synchrony does not affect the relationship between and mean and variance of output, but does affect discriminability. **A**. Across all values of synchrony the mean and variance increase in roughly the same linear pattern; each dot is a stimulus orientation from 0 to 180. At high synchrony values relationship is ultimately violated as the spike count variance plateaus, when the timing variance is smaller than the integration window. Jitter values (in units of ms) are indicated next to the dots that represent the simulation results corresponding to those minimum jitter values. **B**. Each curve shows the spike probability distribution at the preferred orientation. Increasing synchrony shifts the spike count distributions away from the origin, giving more freedom to spread and making adjacent orientations more distinguishable (not shown).

The tuning curve is illustrative to see how well a particular stimulus orientation drives a cortical neuron but by itself it does not convey any context as to how well the cortical neuron transmits information about the stimulus. Synchrony clearly modulates the overall amplitude of this tuning but it is unclear how it modulates the transmission of the underlying stimulus information. The ability of an ideal observer of neural activity to extract meaningful information regarding the features of a visual stimulus depends not only on the shape of the tuning curve, but also on the variability of the cortical response and how this variability changes with the stimulus feature. The statistics of the cortical response are summarized in Figure 5. In Figure 5A, the underlying relationship between the mean and variance of the cortical spike count for all stimulus orientations (each individual dot) is illustrated. The relationship clearly demonstrates an increase of spike count variance relative to spike count mean with a slope of approximately 3, which begins to drop when the input is relatively synchronous (6–10 ms of jitter). The variance begins to drop at extreme levels of synchrony as the decreased amount of added timing variance approaches the size of the integration window of the model, and higher synchrony values effectively make the spike count more deterministic. With respect to the relationship between the mean and variance of the cortical response, experimental results have been variable, exhibiting both sub- and supra-linear variability [33]–[42]. So while the orientation tuning width was relatively invariant to the level of synchrony, as shown in Figure 4C, the increased level of synchrony was accompanied by an increased mean firing rate, and thus an increased variance, the effects of which are not immediately obvious from the perspective of an ideal observer. Figure 5B shows the corresponding spike count distributions for the tuning curves in Figure 4B, for the preferred stimulus orientation (90 degrees). The spike count distribution changed dramatically as input synchrony decreased, with asynchronous inputs pinning spike count distributions at the origin and restricting the discriminability at adjacent distributions, a problem not encountered for highly synchronous inputs. From these results we might qualitatively expect that increasing synchrony would lead to increases in information because synchronization appears to give response distributions a greater range over which to vary with stimulus orientation.

Results from both the mean-variance relationship and the per-synchrony peak spike count response distributions thus lead to conflicting expectations on what level of input population synchrony would drive the maximum amount of information about stimulus orientation. In order to solve this inconsistency we must implement a metric that describes concisely how discriminable different stimulus orientations are and determine the effect input synchrony has cortical information transfer. Fisher information quantifies the degree to which response distributions are discriminable, and thus, provide unambiguous information about stimulus features captured in the response distributions. The simplest understanding of Fisher information in the context of the problem here is that it represents the derivative of the tuning curve with respect to the stimulus orientation; regardless of the underlying firing statistics, the peak Fisher information will occur near orientations where the derivative of the tuning curve is highest.

### Maximum Information Is Modulated by Changes in Input Population Synchrony

**Figure 6:**
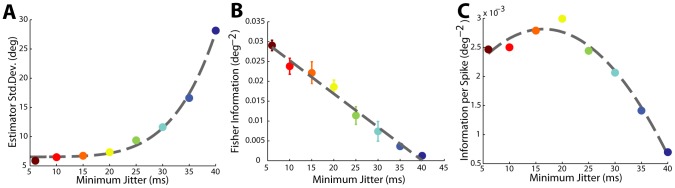
Information efficiency peaks as synchrony increases. **A**. Estimator standard deviation monotonically decreases as the minimum jitter of the input decreases. **B**. The absolute amount of information decreases approximately linearly with increasing minimum jitter. Error bars of ±1 S.D. are shown to illustrate deviations from linearity are not strictly due to random chance. **C**. When weighted by the total output spike count, information efficiency peaks at 15 ms of jitter and then decreases for inputs with smaller amounts of jitter.

We use the peak amount of information across all stimulus orientations for each level of input synchrony as the metric for the capacity for any particular neuron to inform estimations about the stimulus orientation. By itself the absolute amount of information is an unintuitive quantity. With the goal of determining how synchrony changes the capabilities of cortical neurons to decode specific stimulus features, it is more natural to look at properties of the feature estimator. The inverse of Fisher information is the Cramér-Rao lower bound, a theoretical lower bound on the variance of a maximum-likelihood estimator; decreases in this quantity yield estimates that are more precise and have more confidence. Under the assumption that the stimulus orientation estimator is unbiased, lower estimator variance guarantees lower estimator error. Since we could directly calculate Fisher information in our model, we could also determine what this lower bound was, as shown in Figure 6A. The estimator standard deviation decreased nonlinearly with increasing synchrony, covering a range of relatively precise estimates to very imprecise estimates with a notable saturation at around 20 ms of jitter; synchrony higher than this does not yield rapid gains while decreases in synchrony rapidly decrease the estimator precision. As the Fisher information is directly related to the local slope of the tuning curve this qualitative observation was unaffected, in a relative sense, by the discretization of the tuning curve. The raw information decreased approximately linearly with increasing minimum jitter as shown in Figure 6B (error bars are ±1 S.D.). However, as we will show the degree to which this is not linear has important implications for the efficiency of information transmission by the cortical neuron.

From these results, we naively assumed that a strategy which absolutely increased synchrony would always be best as it would always result in increasing stimulus information. As has been noted in other models which bear some similarities to our own [43], there is a metabolic cost to increasing firing rate which can affect the efficiency of some information representations relative to others. In this case, as shown in Figure 6C, when we normalize the absolute amount of information by the number of cortical spikes, it becomes clear that the peak in transmission efficiency occurred at around 15 ms of thalamic jitter, and a quadratic fit had a peak at 16 ms with a clear decrease in information efficiency away from this peak. In previous studies [10]–[12] we identified that pairwise LGN synchrony in response to natural scenes tends to be from 10 to 20 ms as measured by our scale. As noted, this result was consistent across all simultaneously recorded neurons when these neurons were used as sources for single-trial spike times. A few neurons maintained this quadratic relationship between information transmission efficiency and input synchronization at a peak efficiency closer to 25 ms of timing jitter, slightly lower than expected. These results indicate that populations in the LGN are uniquely arranged to be effectively synchronized by a preferred orientation. This synchronization allows information transmission to be more efficient without sacrificing precision in estimating orientation.

### Tuning Width Invariance

**Figure 7:**
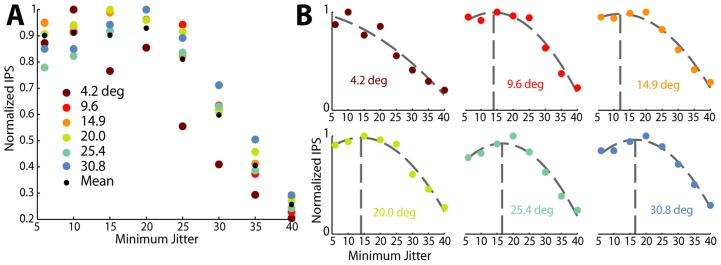
Quadratic efficiency is relatively invariant to tuning width. **A**. When taking the mean (normalized) efficiency curve across the spectrum of reasonable spike count tuning widths (HWHH, degrees), the arragement of optimal efficiencies does not appear to be patterened in any particular way. **B**. When broken out into individual efficiency curves we see that for each tuning width a quadratic polynomial still remains the best fit for most tuning widths. At pathologically narrow tuning curves, we see that higher synchronization is indeed absolutely preferable. We also note that the sigmoid fit to mean data arises, in part, because the peak of the polynomials are distributed over a range and the mean of them produces a roughly constant function below 25 ms of timing jitter.

The results presented so far have demonstrated that information efficiency saturates at levels of minimum timing jitter between 10 and 20 ms, without addressing the effect of tuning width. It is clear from existing literature that there is a wide range of tuning widths that are typically measured in neurons in visual cortex [9], [29]–[32] and these changes are reflected in the width of 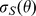 and thus the width of the tuning curve. To investigate the effect of changes in just tuning width we modulated both the minimum timing jitter as well as the tuning width, with the results shown in Figure 7. From 4.1 to 30.8 degrees (HWHH; maroon to light blue dots in Figure 7), which covers the rough range one could expect tuning width to vary, it is clear that the normalized information per spike (IPS) has approximately the same pattern regardless of tuning width. We show normalized information per spike because Fisher information is directly related to the slope of the curve, higher slopes monotonically lead to higher absolute levels of information and as such 4.2 degree and 30.8 degree tuning widths have an order of magnitude difference in their absolute amount of information. The relationship between tuning width and information efficiency is made clearer in the breakouts in Figure 7B for each individual tuning width; with the exception of extremely narrow tuning widths, as the tuning width increases the optimal level of minimum jitter increases but still stays in the range of 10–20 ms. Narrow tuning curves fail to saturate information per spike because very narrow tuning curves effectively contain information about a very small range of orientations and the amount of information is directly related to the diference between baseline and peak firing rates. As an example consider a tuning curve that goes from baseline firing rate to peak firing rate in the span of 2 or 3 degrees (a very narrow tuning curve). In this case higher peak firing rates have a very pronounced affect on the overall amount of information. Since lower jitter always provides higher peak firing rates, narrower tuning curves are always most efficacious at extremely low amounts of jitter. We thus see that the results are valid for a range of primary visual cortex neurons so long as they have tuning widths that are within physiologically measured ranges.

## Discussion

In this work we investigated the role of stimulus-driven synchrony in thalamic populations in the emergence of feature selectivity in primary visual cortex. The complete understanding of this role requires observation of entire thalamic sub-populations which are convergent onto single cortical neurons. Since these populations are too large to record electrophysiologically using current experimental methodologies, we synthesized representative populations from experimental data by randomly choosing recorded trials of neurons from which we could record, when obeying anatomical rules of thalamocortical connectivity [24] (also see below). These populations had an amount of stimulus-driven synchronization that was a direct function of the orientation of a drifting grating stimulus. These synthesized populations allowed us to systematically modulate the underlying spike timing synchrony to investigate the way in which different levels of synchronization affect information transmission. Through a biophysically inspired integrate and fire model that simulates cortical responses, we estimated the resultant cortical orientation selectivity and the corresponding information conveyed about visual stimulus orientation by the cortical response. Ultimately we found that the level of synchronization of the input population had a nonlinear effect on the resulting information contained in the cortical response; higher levels of synchrony led to higher levels of information, but at the expense of a nonlinear increase in firing rate. When taking into account the potential cost of increased firing rate, we found that the most efficient transmission of information was at a level of thalamic synchrony in the range of 10 to 20 ms.

It is important to note that the synchronization of neurons has been widely studied in a number of different contexts. Notably, synchronization of neurons across cortical columns has been previously reported in the visual cortex, proposed as a means to form relationships across regions of the visual field [44]. Additionally, in the context of convergence and divergence of retinal afferents projecting to the LGN, precise correlations have been observed across geniculate neurons that were present in the absence of stimulus driven correlations, and were attributed to the projections of common retinal ganglion cell inputs [13]. In contrast, the current study (and previous studies from our group [11], [23]) specifically examines the role of stimulus driven synchronization/correlation of neuronal firing in the visual thalamus. Our previous investigations have shown that many neurons in the LGN do not exhibit appreciable noise correlations [11]. The focus here is thus on the relationship between the visual input and the resultant synchronization of firing activity across geniculate ensembles, a requisite for robust activation of the downstream cortical neurons to which they project. In the most general case, however, as described in Gray et al. [44], the propagation of neuronal signals would involve a combination or interaction between the synchronization due to ongoing spontaneous activity and the stimulus-driven synchronization due to coordinated activation of neurons sharing the same topology and feature selectivity. Such a “from-any-source” view of synchronization carries with it the possibility that neurons with receptive fields from disparate regions of the visual field could be synchronized by spatially correlated stimuli. For example two very spatially distant LGN neurons could be simultaneously activated by either two unrelated objects or one very long bar of light; synchronization due to these origins are not considered in this model. It is important to note that we explicitly consider only recordings from spatially localized populations, as widely-spaced LGN units do not converge at the same cortical target.

The emergence of orientation selectivity in primary visual cortex is perhaps the most well-studied example of cortical computation to date. As a result, there have been a large number of modeling studies seeking to capture the mechanistic explanation for the primary observation of orientation selectivity, and also to capture a number of related, and more complex functional properties (e.g. contrast invariant orientation tuning, cross-orientation suppression, etc.). Given that there is little if any dispute as to the role of direct feed-forward geniculate input to cortical layer 4 in establishing the basic orientation preference for cortical neurons, models of orientation selectivity have invariably been constructed around a backbone of thalamic input. Although the model from Ringach introduced structured synaptic weightings and connectivity probabilities of thalamic inputs to cortex as a key model element [5], the majority of other models assume relatively simple feedforward excitation structure and differ primarily in the relative strengths of the feedforward or intracortical inhibition [3], [4], [26]–[28], [45], [46]. A specific limitation of most of these previous models is that they explicitly do not directly involve electrophysiological data as thalamic input. For example, one class of models use simulations of thalamic or retinal responses based on the stereotypical difference-of-Gaussians representation of center-surround receptive fields [3]–[5], [45], [46], while others rely on assumed or derived cortical conductances or membrane potential but not on actual thalamic input [26]–[28]. The large majority of previously published models also assume that sinusoidal inputs (i.e. drifting gratings) elicit sinusoidal thalamic responses and that the cortical membrane potential itself is perfectly sinusoidally modulated (as in [9] or [26], [45]). Dating back to the early 1980s there was the observation that drifting sinusoidal gratings produced asymmetric LGN response PSTHs (i.e. a sharp peak at the onset of the stimulus followed by a long tail of decaying response) [47]–[49] and more recently we have directly analyzed the effects of this synchrony in the context of cortical orientation and direction selectivity [23]. We assert that the precise timing and stimulus-driven synchronization of thalamic inputs serves a prominent role in the thalamocortical circuit and in the emergence of cortical feature selectivity.

It is important to note that most, if not all, existing models designed to capture the mechanism behind cortical orientation selectivity rely on spatial arrangements of projecting thalamic inputs that in some cases exceed those observed experimentally [24]. More specifically, the relevant measure for thalamic input is the aspect ratio of the scatter of thalamic receptive fields that form the input to a single cortical layer 4 neuron. Recently, Jin et al. experimentally observed thalamic clusters and showed that the thalamic input to cortical orientation columns has receptive fields that are highly overlapped [24]. Because the scatter of the thalamic receptive fields covers 2.5 receptive field centers in visual space, the average layer 4 cortical neuron should have a maximum aspect ratio of 2.5∶1. The thalamocortical model from Somers et al. was built on an aspect ratio of 3∶1 [3], whereas the model from McLaughlin et al. was built on an aspect ratio of 4∶1 [4]. Similarly large aspect ratios are apparent from the Kayser et. al. model and Finn et. al. models, with ratios approximately 6∶1 and 2.5∶1 respectively [26],[46]. It is clearly the case that inhibitory mechanisms play a significant role in the shaping of the cortical feature selectivity [2], and would only serve to further refine the selectivity established by the direct feedforward thalamic input shown here. Many of the above-mentioned models differ from our presentation here in that they include OFF-center sub-populations in the thalamic population, most commonly offset from the ON-center population as would be implied by the common Gabor-type simple cell receptive field. To keep the model relatively straightforward and simple, we have chosen to focus on just ON-center populations.

The majority of existing models were optimized to explain extra-classical effects of cortical receptive fields with a particular focus on the contrast invariance of cortical tuning width and as such constructed mechanisms specific to this issue. Specifically, it has been widely observed that although peak cortical firing rates are strongly dependent upon stimulus contrast, cortical orientation tuning is largely invariant to stimulus contrast (for review, see [2]). This observation called into question the purely feedforward model of cortical orientation selectivity [2]. Subsequent models augmented the feedforward thalamic input with inhibitory feedforward connections [26] or cortico-cortico inhibition [46] or some combination [2], [3]. We have previously shown that thalamic synchrony is largely unaffected by stimulus contrast [11], and the cortical tuning based on thalamic synchrony is also contrast invariant. The model we have proposed here thus potentially demonstrates a completely feed-forward explanation for contrast invariance. For a fixed minimum jitter amount, as the underlying LGN firing rates across the entire population are modulated by changes in the stimulus contrast, the peak induced firing in the cortical neuron rises and falls. Since the changes in LGN firing are correlated across the LGN population, the synchrony across such a population (with particularly focus on the relationship between stimulus orientation and the synchrony) remains unchanged as a function of stimulus contrast. As demonstrated in Figure 4B for the span of biophysical levels of preferred orientation population synchrony (∼5 to 20 ms), the tuning width of the cortical neuron does not change, indicating that changes in the degree of underlying synchrony do not change the tuning properties. Although the results are not presented here directly, the combination of past and present results suggest that changes in the LGN population response (i.e. the population becomes less active in general) lead to a decreased or increased peak cortical response but the tuning curve widths will be invariant to stimulus contrast.

We used Fisher information as a measure of the efficacy of cortical neurons in representing stimulus features (orientation) in response to changes in the synchrony of an input population. Specifically, we used the peak Fisher information irrespective of the orientation at which the peak occurs. Contrary to previous investigations [50]–[52] in which the absolute value of the Fisher information was used as an important measure of the performance of neural populations, here we sought to capture the relative effects of varying degrees of thalamic synchrony on the information conveyed by a single recipient cortical neuron target. In this case, we assumed that the Cramér-Rao lower bound need not be met and that whatever bias causing deviations from the lower bound is consistent across all simulation conditions. We ensure this by using the same input data and model structure for all conditions so that we can compare relative levels of information across different synchrony conditions for a single neuron. Although this is a simplification of the true amount of information (and indeed no single neuron will saturate this lower bound), in either case the absolute information was consistent with previous studies utilizing experimental cortical data. Yarrow et. al. [52] computed Fisher information for both real and simulated neural populations and found an information level which was approximately consistent with the findings presented here (see their Figure 4 as well as [51] Figure 3, with axes in [52] helping in the conversion from SSI bits to Fisher Information in units of 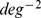). This assumption ultimately only affects the reporting of estimator standard deviation (as in Figure 6A) which was not the primary result of the work.

It is also important to note that the application of Fisher Information to cortical tuning curves has deeper roots in estimating cortical population response information transmission. Past work [53]–[57] has in general used constructions where a collection of identical cortical neurons have preferred orientations that uniformly span the orientation spectrum (0 to 360 degrees). In this study we considered only a single neuron in the population. We claim, though, that results which demonstrate information in a single neuron at all stimulus orientations are fundamentally identical to results which demonstrate information in a population at a single orientation. As long as we assume every neuron in the cortical population is conditionally independent, for the questions we ask these two formulations are fundamentally interchangeable. As identified in [54] under the assumption that each cortical neuron in this population is independent, then at every stimulus orientation the overall Fisher information is
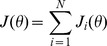
Further, in the case that every neuron in the population is also assumed to be identical in response properties, then we can modify the above to read (for any choice of 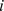)
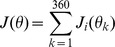
It is clear though that not all cortical tuning curves are identical and the absolute amount of information is strongly negatively correlated with tuning width. Using this fact as inspiration, we show in Figure 7 that the optimally efficient level of input timing jitter is widely insensitive to the tuning width of the cortical neuron. In this case, even if a cortical population is composed of non-identical independent neurons, each neuron, as well as the population as a whole, will be optimally efficient as long as the thalamic input is synchronous to the 10–20 ms level (thus implying we need no longer assume neurons within the population have identical, but shifted, tuning curves). If we further consider the effects of correlated variability, as in [55], then we can no longer assume the units are independent. Regardless of whether the correlated variability increases or decreases the absolute amount of information (and neither is guaranteed), correlated variability would raise or lower the response rate of the individual neurons in a coordinated manner. Since again our metric is one of relative comparisons, the results presented here are expected to be invariant to correlated variability in the sense that the efficiency of any single neuron may decrease, the peak efficiency will still occur between 10–20 ms (which would still be true for all neurons in the cortical population). Thus our findings directly translate to cortical populations regardless of the independence and homogeneity of tuning properties of the component neurons.

In previous studies of timing precision of individual thalamic neurons [10] and across thalamic pairs [11] in response to natural scenes, we have reported characteristic timescales on the order of 10–20 ms. In these previous studies, measures were taken across long segments of natural scene movies, representing the aggregate of instantaneous firing events whose timing precision clearly varies on an event-by-event basis [12], [58]. The instantaneous synchronization of firing activity across a sub-population of neurons in the context of natural scenes is undoubtedly a complex function of the local properties of the scene, including spatial frequency, temporal frequency, and orientation of the local spatial structure. It is thus the case that the 10–20 ms average timescale reflects a distribution of synchronous events, spanning from synchrony on just a few milliseconds to more asynchronous firing over a timescale of 10€s of milliseconds, unlikely to drive the cortical target. Here, we report that in the context of the modulation of thalamic synchrony through visual stimulus orientation with drifting sinusoidal gratings, the most efficient level of thalamic synchrony in conveying relevant information to cortex is in the 10–20 ms range. This means that, on average, amongst natural scenes and all their various features, the thalamic neural response is tuned to maximize the efficiency of information transfer to the cortex (similar to [22]). As we have investigated only the effects of orientation changes on synchronization and feature selectivity, we expect that this result implies that information efficiency will be similarly optimized for other visual features such as spatial and temporal frequency. Furthermore, it is possible that synchronization optimizes information transmission in entirely different sensory systems, given previous findings in the somatosensory system [59].

## Materials and Methods

### Ethics Statement

Surgical and experimental procedures were performed in accordance with United States Department of Agriculture guidelines and were approved by the Institutional Animal Care and Use Committee at the State University of New York, State College of Optometry.

### Surgical Preparation and Electrophysiological Recordings

The experimental data collection has been previously described [23]. Briefly, single-cell activity was recorded extracellularly in the lateral geniculate nucleus (LGN) of anesthetized and paralyzed male cats, with a total of three animals. As described in [60], cats were initially anesthetized with ketamine (10 mg kg^−1^ intramuscular) and acepromazine (0.2 mg/kg), followed by propofol (3 mg kg^1^ before recording and 6 mg kg^−1^ h^−1^ during recording; supplemented as needed). A craniotomy and duratomy were performed to introduce recording electrodes into the LGN (anterior, 5.5; lateral, 10.5). Animals were paralyzed with vecuronium bromide (0.3 mg kg^−1^ h^−1^ intravenous) to minimize eye movements, and were artificially ventilated. Using a seven-electrode matrix, layer A geniculate cells were recorded extracellularly. The multielectrode array was inserted into the brain to record from iso-retinotopic lines across the depth of the LGN, using an angle of 25–30 degrees antero-posterior, 2–5 degrees lateral-central. To a multielectrode array (with inter-electrode separation of 254 µm) we attached a glass guide tube with an inner diameter of 300 µm. As the elevation axis is better represented in LGN than the azimuth axis, some of the populations of LGN receptive fields showed greater lateral than vertical scatter in the visual field [61]. Layer A of LGN was physiologically identified by performing several electrode penetrations to map the retinotopic organization of the LGN and center the multielectrode array at the retinotopic location selected for this study (5–10 degrees eccentricity). While recording, the RASPUTIN software (Plexon, Dallas, TX) was used to capture voltage signals after being amplified and filtered. We isolated single units by independently moving each electrode and the resulting units were spike-sorted online and verified offline using a commercially available algorithm (Plexon, Dallas, TX). Cells were eliminated from this study if they did not have at least 1 Hz mean firing rates in response to all stimulus conditions. Cells were classified as ON or OFF according to the polarity of the receptive field estimate.

### Visual Stimulation

For each cell, visual stimulation consisted of multiple repetitions of a drifting sinusoidal grating at 0.5 cycles/degree, at either 100% or 64% contrast. The direction of the drifting grating was varied. The orientation of a particular drifting grating was one of eight possible values: 0, 45, 90, 135, 180, 225, 270, 315 degrees. The convention was that a vertically oriented grating drifting rightward was referred to as 0 degrees, a horizontally oriented grating drifting downward was referred to as 90, and so on. The temporal frequency for all datasets was 5 Hz or 4 Hz. The spatial resolution for the drifting gratings was 0.0281 degrees per pixel. All stimuli were presented at a 120 Hz monitor refresh rate.

### Generating LGN Population Activity for Model Input

Biophysiological levels of LGN population synchrony were measured from multiple sets of simultaneous electrophysiological recordings (between 5 and 7 neurons were recorded simultaneously). A cortical neuron is thought to receive approximately 30 LGN inputs [8] but these neurons are substantially more densely arrayed than we can reasonably hope to record with penetrating electrodes. Population response estimates were achieved by expanding the simultaneous recorded neurons into a population of 30 neurons by replicating the recorded responses and then shifting to a new visual location, restricted within the visual space bounded by the original receptive field locations. This restriction resulted in a population that has a receptive field center diameter distribution that is consistent with [24] (see Figure 1D). To create the population random shifts were allowed in both the vertical and horizontal directions (i.e. the major and minor axes of the population) but the restrictions placed by the original population layout often required greater shifts along one or the other axis. For the example in Figure 1C, the shift restrictions resulted in a visual space coverage of approximately 5 degrees (horizontal) by 2 degrees (vertical). Shifting responses required knowledge of the timing difference in excitation between the old and the new location, defined as the shift latency. The replicated input spike trains occurred in response to sinusoidal gratings and, due to the regularity in the stimulus, the shift latency was relatively easy to calculate. This shift latency was estimated simply by measuring the timing latency between the maximum excitation at the centroids of the receptive fields at both the original location and the shifted location
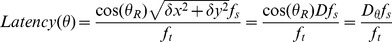
where 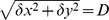 is the center to center separation of the original and shifted locations, 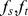 represent the spatial (cycle/deg) and temporal (Hz) frequencies (fixed) of the stimulus itself, and 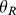 is the angle between the axis connecting the two receptive fields and a line from the shifted location perpendicular to the oriented stimulus bar. A graphical representation of this is in Figure 1D. Each newly created neuron is assigned a random trial from all recorded trials of the original neuron and the shift latency value is added to all spike times within that chosen trial. For the representation of this process in Figure 1E each neuron received a trial from the appropriate stimulus orientation. As the overarching cortical model, though, expands to a much larger set of orientations than originally recorded from, for consistency each newly created neuron was assigned a trial from the recordings performed with a stimulus at a 0 degree orientation. This allows us to preserve the baseline across-neuron timing changes, while capturing the stimulus-driven timing modulations with our 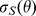 parameter, discussed below.

The model was constructed such that all input synapses to the cortical neuron have equal strength and no particular synaptic location (i.e. along the dendrite or at the soma), and accordingly the source of the spikes from within the LGN population has no effect on the actual model output. Since this is the case, we can estimate the input population auto-correlation by collapsing all LGN spike times into a single vector. The auto-correlation is then calculated by subtracting each spike time from all other spike times and calculating the histogram of these pair-wise interspike intervals. Synchronous populations will have a much higher proportion of small intervals (neglecting stimulus periodicity) than asynchronous populations. The auto-correlations are also appropriately normalized to be between 0 and 1. To smooth the auto-correlation and eliminate correlations caused by the periodicity of the input, a Gaussian was fit to the central 200 ms lags in the correlation. We use timing jitter as a metric of synchrony, which is determined by normalizing the Gaussian fit and locating the lag at which this curve is equal to 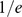. To relate this number to the PSTH timing jitter (i.e. combined population timing jitter) we must divide by two (see Supplemental in [10] for a complete description). In brief, we define a value 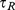 which is the “response timescale”. This value is equal to the latency at which the auto-correlation equals 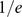. By construction this has the relationship that 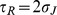, where 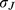 is the timing jitter in the PSTH, our value of interest. This process was performed for all stimulus orientations (in order to maintain phase and timing differences that arise from differences in neuron properties and not just spatial relationships) to describe timing jitter as a function of stimulus orientation. This function was calculated multiple times for different randomly generated populations to estimate the variance that is created by choosing either different visual locations for the component neurons or choosing different recorded trials to represent the neurons in the population.

The observed timing variability in spike times across the population is composed of two aspects; intrinsic neural variability and variability caused by the interaction between the stimulus and the population organization. Our model captured the intrinsic variability by using spike times that were recorded in vivo. On the other hand, while the grating stimulus always evokes firing in the thalamic neurons the timing differences in spike times from neuron to neuron will vary according to orientation of these gratings and the arrangement of the population itself. We capture this stimulus-evoked timing variability in a parameter 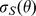. This parameter, as a function of stimulus orientation, was manually calibrated such that when used with recorded data we could reconstruct the exact plot shown in Figure 2C. This procedure allows us to capture both the intrinsic and stimulus-evoked sources of spike timing variability even at orientations for which we were not able to collect data.

### Integrate and Fire Model of Direct Synaptic Input to a Cortical Layer 4 Neuron

All simulations and computations were performed in the Matlab programming language (Mathworks, Inc., Natick, MA) using a 64-node grid computer. The integrate and fire model [62], illustrated in Figure 3A, takes spiking activity from the simulated LGN population as input and outputs cortical membrane potential and the associated cortical spike times. It was assumed that each synapse has equal strength. To create the synaptic input current, an exponentially decaying EPSC of defined amplitude (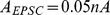) and time constant (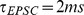) was generated for all spike times in the input LGN population. The EPSCs were summed linearly across all LGN inputs to create a single current input at every simulation time point. The cortical membrane potential was modeled with the following first-order differential equation:
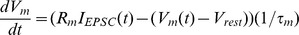
where 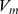 is the membrane potential, 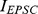 is the total synaptic current, 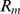 is the membrane resistance (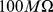), 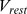 is the resting potential (−70 mV), and 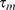 is the membrane time constant (2 ms). The integration was performed using the forward euler method with a step size of 0.05 ms; since the step size is significantly smaller than any other temporal dynamics or spike timing precision use of a simple euler method is sufficient. When 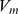 exceeds the threshold membrane potential (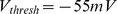), a cortical spike is generated by setting the instantaneous potential to 0 mV followed by a 3 ms refractory period at the reset potential of −65 mV. These values are similar to those we have used previously for similar models [22], [23]. An analysis was performed to determine the approximate sensitivity of the model to each of the above indicated parameters. In general the model is sensitive to parameters which modulate the strength (or efficacy) of input spikes relative to the generated EPSC. Thus the model is sensitive to the EPSC amplitude (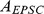; effective values 0.05 to 0.1 nA within acceptable ranges) and the EPSC decay (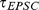; effective values 2 to 5 ms) while being robust to changes in threshold and reset potentials (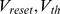). Sensitivity manifests itself as a change between one of three states; impoverished cortical firing, sufficient cortical firing, and strong cortical firing. Impoverished firing results in a peak information per spike (see Figure 6C) at very low jitter values (as this maximizes the chance to get any spikes) and strong firing demonstrates no discernible peak information per spike for any particular jitter value (as it results in very wide tuning curves).

### Fisher Information

Taking the perspective of an ideal observer, we approximated the capability of the observer to discriminate between visual stimulus orientations based on cortical activity alone. More specifically, the Fisher information 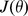 [54]–[57] at each orientation 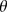 captures the discriminability between 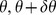

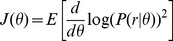
where the expectation is taken with respect to 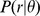. In the case that the probability is zero, we set 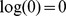. We calculated the derivative numerically using increments of 1 degree which was the resolution at which the simulations were performed. To reduce the results of this calculation to a single descriptive value, we report the estimator minimum standard deviation, which is related to the Fisher information through the Cramér-Rao lower bound (assuming the estimator is unbiased):
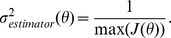
As a metric of efficiency with which the cortical output conveys information about the stimulus, we divide the peak output information by the peak spike count with the goal of identifying how much each individual spike contributes to the overall information; higher values indicate each spike is more efficient at conveying information about stimulus features. This established a penalty for higher firing rates, realizing that there is a metabolic cost to generating action potentials.

### Estimating Response Distribution

Response distributions of the cortical firing rate were estimated based on the simulated data, in order to calculate the Fisher Information. The firing rate varied as a function of 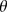 and the distributions are given by 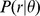. The data were explicitly fit to a Poisson distribution, consistent with previous findings [33]–[42] as well as explicitly verified for appropriate fitting against our own data:
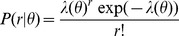
To generate an accurate estimation of the response distributions at a minimum 250 simulation trials were run, with more trials providing no significant change in the estimated distributions. Note that the distributions change as a function of stimulus orientation, as indicated by 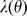. Further, in order to create a smooth description of Fisher information it was necessary that the response distributions be smooth functions of 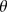, as even minor fluctuations in the 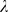 parameter get magnified by differentiation and squaring. To alleviate this, 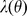 was smoothed with a Gaussian fit which was empirically verified to describe 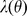 well.
